# Deciphering the molecular origin of the 19.3 eV electronic excitation energy of H_3_^+^

**DOI:** 10.1039/d5sc09067a

**Published:** 2026-01-20

**Authors:** Josene M. Toldo, Jakob K. Staab, Eduard Matito, Cina Foroutan-Nejad, Henrik Ottosson

**Affiliations:** a Department of Chemistry –Ångström, Uppsala University 751 20 Uppsala Sweden henrik.ottosson@kemi.uu.se; b Université Claude Bernard Lyon 1, ENS de Lyon, CNRS, Laboratoire de Chimie, UMR 5182 69342, Lyon Cedex 07 France; c Department of Chemistry, The University of Manchester Oxford Road Manchester UK; d Donostia International Physics Center (DIPC) 20018 Donostia Euskadi Spain; e Ikerbasque, Basque Foundation for Science 48009 Bilbao Euskadi Spain; f Institute of Organic Chemistry, Polish Academy of Sciences Warsaw Poland

## Abstract

The trihydrogen cation, H_3_^+^, is unique in the Universe. It serves as the primary proton reservoir, driving essential astrochemical reactions, and it functions as a thermostat for giant gas planets. H_3_^+^ has also a remarkably low photodissociation rate, explained by its exceptionally high first electronic excitation energy (19.3 eV), which is well above the ionization energy of the much more abundant monohydrogen (13.6 eV). Herein we reveal that the key factors behind the high excitation energy of H_3_^+^, and thus, its astrophotochemical inertness, are: (i) aromatic stabilization in its electronic ground state, (ii) antiaromatic destabilization in its first excited state, and (iii) a high nuclear-to-electronic charge ratio (+3 *vs.* −2). Through comparisons with analogous (isolobal) π-conjugated carbocations, we find that ground state aromatic stabilization plus excited state antiaromatic destabilization raise the excitation energy of H_3_^+^ by 4.8–6.0 eV. This means that for H_3_^+^, the excited state antiaromatic character (which normally leads to high photoreactivity) contributes to its astrophotochemical inertness. Thus, only with the increase in excitation energy due to ground state aromaticity plus excited antiaromaticity can H_3_^+^ act as a thermostat for giant gas planets and as a proton reservoir that drives astrochemical reactions, thereby fulfilling its unique role in space.

## Abstract 2

The trihydrogen cation, H_3_^+^, is unique in the Universe. One of the features that enable its uniqueness is an antiaromatic destabilization in its lowest electronically excited states.

## Graphical abstract

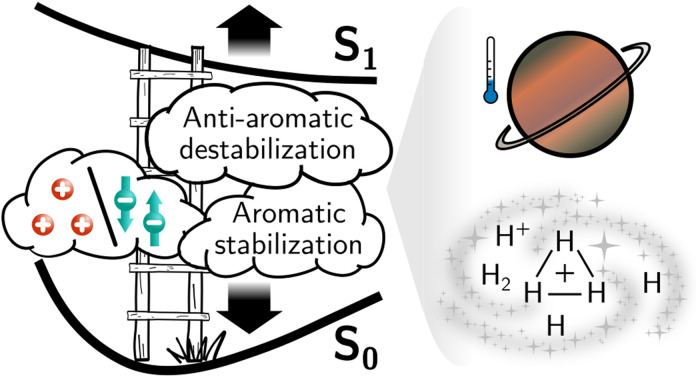

## Introduction

**Fig. 1:**
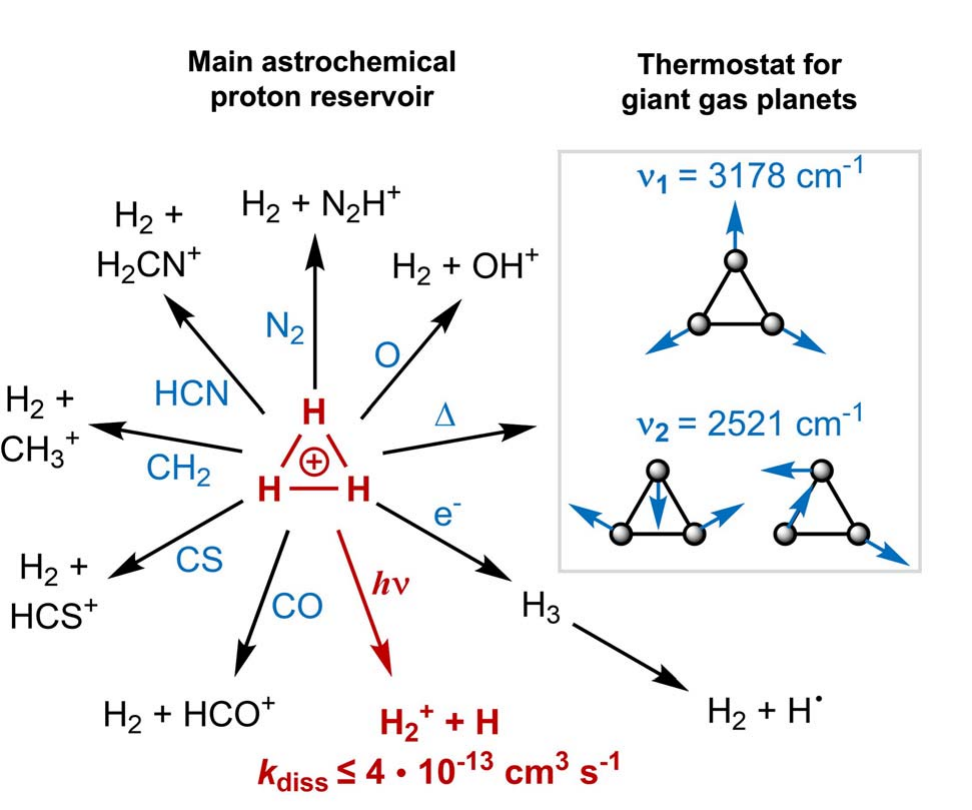
A summary of the functions of H_3_^+^ in space, with its role as (i) primary proton reservoir initiating a number of core astrochemical reactions (typical rate constants for these reactions are ∼10^−9^ cm^3^ s^−1^), and (ii) as a thermostat in the upper atmospheres of giant gas planets ensuring that their temperatures do not exceed a threshold.

Triangular H_3_^+^ is the most abundant polyatomic ion in the interstellar medium, where it functions as the primary interstellar acid, initiating reactions that lead to more complex molecules (Fig. 1).^1–3^ It further acts as a thermostat (coolant) in the upper atmospheres of giant gas planets,^4^ and it has been postulated that it even could have functioned as a coolant in the primordial gas (though with a different mechanism than in the giant gas planets).^5^ With three H atoms, it is the smallest molecule that exhibits aromaticity (σ-aromaticity),^6–9^ a stabilizing molecular property.^10^

**Fig. 2:**
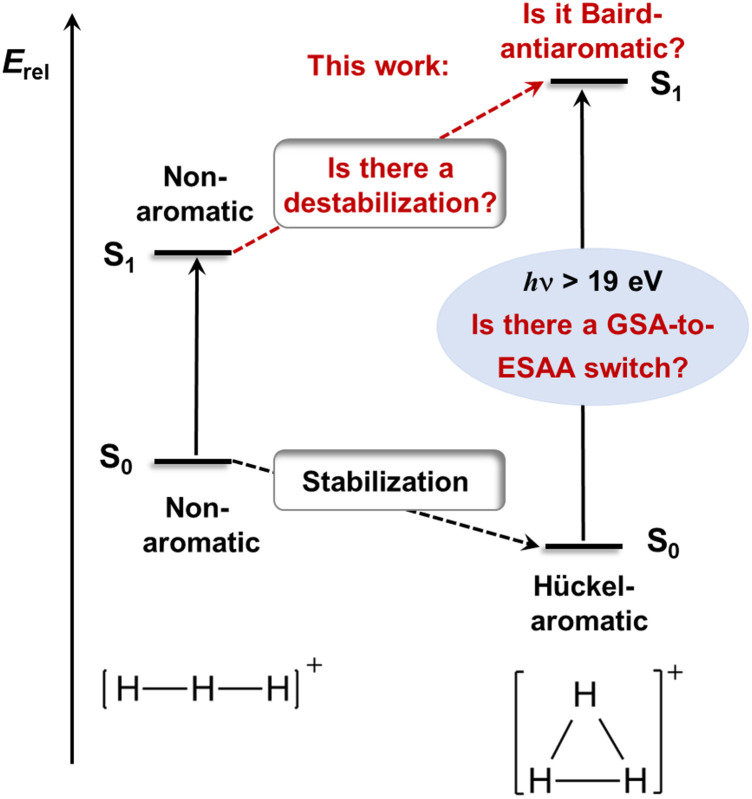
Tentative changes from the nonaromatic character of linear H_3_^+^ in its S_0_ and S_1_ states to the equilateral triangular H_3_^+^ with a stabilizing Hückel-aromaticity in S_0_ (GSA = ground state aromaticity) and a destabilizing Baird-antiaromaticity in S_1_ (ESAA = excited state antiaromaticity). Factors related to our hypothesis and explored herein written in red, while known factors in black.

The first electronically excited singlet states of H_3_^+^, the doubly degenerate 1^1^E′ states at the equilateral triangular structure (*D*_3h_ symmetric), are of exceptionally high vertical energy (19.3 eV, Fig. 2),^11^ and they are dipole-allowed and dissociative.^12–14^ Despite this, astrochemical databases list a photodissociation rate of 4 × 10^−13^ s^−1^ or lower,^13,15^ whereby it is among the astrochemical species with lowest photodissociation rates.^16,17^ The reason is that the much more abundant H and H_2_, with ionization and excitation energies at 13.1–15.4 eV,^1,18^ shield H_3_^+^ from high-energy irradiation in the interstellar medium (ISM).^12,13,19,20^ In the ISM, H_3_^+^ degrades unimolecularly only when exposed to electrons ejected from other molecules or atoms upon cosmic ray ionization.^1,13^ Indeed, direct photodissociation only occurs when H_3_^+^ is rovibrationally excited, whereby the electronic excitation energy decreases down to 4.9 eV.^12,13^ Had it been more prone to photodissociate, this would have impaired its astrochemical and astrophysical functions, and thus, been detrimental to the development of the Universe as we presently know it. However, the molecular origins of its very high vertical excitation energy remain unknown and have never been addressed earlier. With such fundamental knowledge in hand it should also be possible to pinpoint other species of astrochemical relevance that may also exhibit unusually high first electronic excitation energies.

H_3_^+^ in its electronic ground state (S_0_) has a *D*_3h_ symmetric structure^11,21–23^ with σ-aromatic character,^6–9^ in line with Hückel's 4*n* + 2 rule (*n* = 0, 1, 2…) as it has two σ-electrons. We now hypothesize that the counter-concept, antiaromaticity,^10^ is relevant for its first excited state of singlet multiplicity (S_1_), and thus, its excitation energy (Fig. 2). In this context, the analogous cyclic and fully π-conjugated hydrocarbons (*i.e.*, annulenes) in their lowest excited triplet states (T_1_) of ππ* character follow Baird's rule,^24–28^ which tells that molecules with 4*n* + 2 π-electrons are antiaromatic and destabilized in these states while those with 4*n* are aromatic and stabilized. Although derived for the T_1_ state, the rule can often be extended to the lowest ππ* excited state of singlet multiplicity. Thus, the exceptionally high excitation energy of H_3_^+^ may stem from σ-antiaromatic destabilization in S_1_ (Fig. 2) combined with σ-aromatic stabilization in S_0_, *i.e.*, a switch in character from ground state Hückel-aromaticity (GSA) to excited state Baird-antiaromaticity (ESAA), a GSA-to-ESAA switch in character.

Accordingly, we now investigated if the lowest excited states of H_3_^+^ are antiaromatic and based this assessment on quantum chemical calculations of various (anti)aromaticity descriptors. Is ESAA the factor that leads to the very high excitation energy or are there other factors? Furthermore, can knowledge gained through this investigation impact on other parts of astrochemistry? The H_3_^+^ cation has a 3-center 2-electron bond, a type of bonding it shares with nonclassical carbocations such as the vinyl (C_2_H_3_^+^) and ethyl (C_2_H_5_^+^) cations,^29–37^ species which are also of astrochemical relevance.^38,39^ One can thus argue that the findings herein can be useful to understand the astrophotochemical features of these species as well.

Computations of H_3_^+^ were run at the EOM-CCSD level for all species, which for H_3_^+^ corresponds to a full configuration interaction (FCI) calculation, *i.e.*, a numerically exact solution of the Schrödinger equation. These computations were performed using the aug-cc-pVTZ basis set of Dunning. For further computational details, see the Computational methods section and the SI.

## Results and discussion

Herein, we first present and discuss results of the potential energy surfaces of the S_0_ and lowest few singlet excited states, followed by assessments of the (anti)aromatic characters of these states. This allows us to get a first tentative link between the excited state surface profiles and ESAA alleviation, yet, other factors that can impact on the lowest excitation energy of H_3_^+^ are also identified and explored. Towards the end we compare with the analogous (isolobal) carbocations, which allows us to estimate the energetic component of a GSA-to-ESAA switch in character on the vertical excitation energy of H_3_^+^.

### Potential energy surfaces

**Fig. 3:**
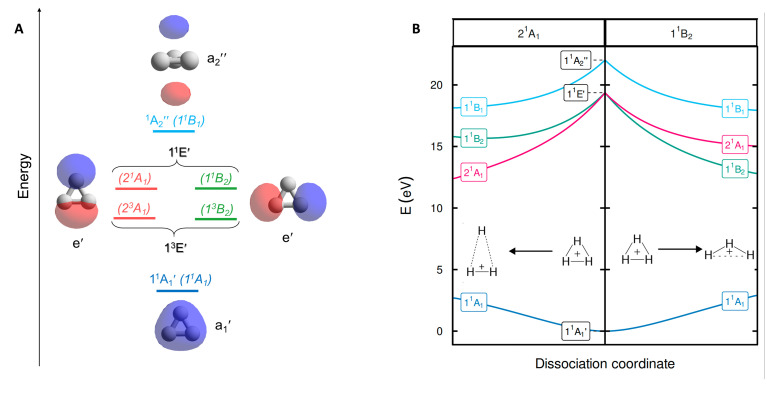
(A) The three valence MOs (
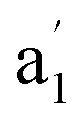
 and e′), the Rydberg orbital 
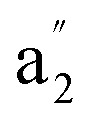
, and the labels of the electronic states within the *D*_3h_ (normal print) and *C*_2v_ (in parenthesis and italics) point groups. (B) Potential energy surface profiles of H_3_^+^ from the *D*_3h_ symmetric S_0_ equilibrium geometry along the minimum energy path of the 2^1^A_1_ state keeping *C*_2v_ symmetry to an acute isosceles triangle (left), and along the minimum energy path of the 1^1^B_2_ state to an obtuse isosceles triangle (right).

According to our computations, the H–H distances of equilateral triangular H_3_^+^ in S_0_ are 0.875 Å which is very close to the earlier computed value of 0.873 Å found with variational Born–Oppenheimer theory using explicitly correlated Gaussian functions.^11^ The degenerate 1^1^E′ states appear vertically 19.28 eV above the S_0_ state (Fig. 3A), again very similar to the reference value of 19.33 eV.^11^ Expansion of the basis set beyond aug-cc-pVTZ has a negligible effect on the vertical excitation energy (19.2883 eV with FCI/aug-cc-pV5Z). The transition is symmetry allowed with an oscillator strength *f* = 0.562, yet, as it is well above the ionization energy of H (13.6 eV), the excitation has exceptionally low probability.

After excitation, in *C*_2v_ symmetry, the 1^1^E′ states split into the 2^1^A_1_ and 1^1^B_2_ states (Fig. 3B), where 2^1^A_1_ as S_1_ dissociates to H_2_^+^ + H while 1^1^B_2_ as S_1_ leads to 2H(1s) + H^+^.^14^ These dissociative forces can be understood as a result of the Jahn–Teller theorem.^40^ Along *D*_3h_ symmetric geometries, the degenerate ^1^E forms a conical intersection seam, which is lifted by symmetry-breaking nuclear distortions along a pair of E type vibrations, leading to electronic stabilization. We recognize that extensive vibronic coupling effects influence the excited state spectrum and that these, as well as different conical intersections, strongly impact on photochemical dissociation dynamics of H_3_^+^, as has already been explored in previous studies based on highly accurate potential energy surfaces.^13,41–46^ The current work, however, does not attempt to quantitatively model neither spectroscopic data nor photodissociation rates. Instead, the objective of our study is to elucidate the origin of the unusually high electronic excitation energy of H_3_^+^, which hitherto is totally unexplored.

The first triplet states (the degenerate 1^3^E′) have vertical excitation energies of 14.87 eV (Table S1) and exhibit similar dissociative behaviors as 1^1^E, in line with earlier findings.^47^ Lastly, the higher energy 
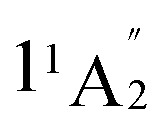
 state in *D*_3h_ symmetry is a dissociative second-order saddle point with H–H distances of 1.620 Å. As this state involves an excitation from an 
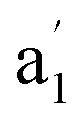
 orbital to a nonbonding Rydberg-type orbital 
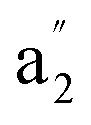
 (Fig. 3A), only one electron remains in a bonding molecular orbital (MO), which cannot counterbalance the electrostatic repulsion between three protons. For further results and discussions on this state, see Sections S1 and S2 of the SI.

### Probing excited state antiaromaticity

The aromatic or antiaromatic characters of the various electronic states were determined comprehensively through use of energetic, electronic, and magnetic (anti)aromaticity descriptors (for details see Section S3 or ref. 10). Thus, we analyze changes in three of the four aspects of aromaticity, with the geometric aspect being impossible to assess due to the dissociative nature of the excited states. The energetic aspect is determined through the extra cyclic resonance energy (ECRE).^48,49^ To measure electron delocalization,^50^ we use the two-center delocalization index (DI)^51,52^ and the normalized multicentre index (MCI^1/*n*^)^53^ that reflect the aromatic character. The magnetic response properties are explored by magnetically induced current densities (MICDs), and also by nucleus independent chemical shifts (NICSs).

**Fig. 4:**
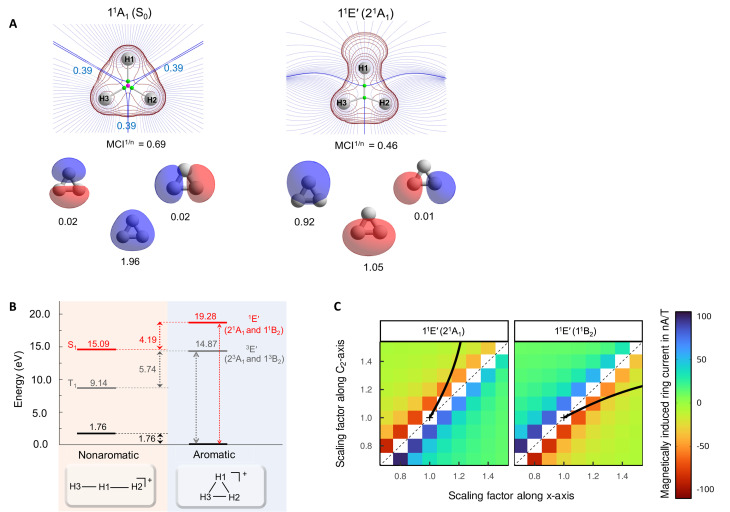
(A) Topological analysis of the electron density, 2D Laplacian of the electron density (in red), and natural orbitals (with populations) for the S_0_ and 1^1^E′ states, the latter labelled as 2^1^A_1_ and 1^1^B_2_ in *C*_2v_ symmetry. The rays of the basins drawn in blue and density gradient lines in purple. MCI^1/*n*^ values (computed using Becke-rho's partition)^56^ are given below the Laplacian plots of the electron density. (B) Vertical excitation energies and relative energies of H_3_^+^ at, respectively, *D*_∞h_ and *D*_3h_ symmetries. (C) Magnetically induced ring currents for the 2^1^A_1_ and 1^1^B_2_ states which stem from the 1^1^E′ states upon geometric distortions to *C*_2v_ symmetric structures. The scaling factors reflect how large this distortion was (the value 1.0 represents the H–H bond lengths of the S_0_ equilibrium geometry). The *C*_2_-axis indicates distortions in the direction of forming an acute isosceles triangle (moving H1 atom) and the *x*-axis distortions along an obtuse isosceles triangle formation (increasing the separation between H2 and H3).

Although assessed in earlier reports,^6–9^ the values for σ-aromaticity of the S_0_ state are discussed briefly in order to contrast the antiaromatic character of the excited states (*vide infra*). H_3_^+^ has two electrons and forms a 3-center 2-electron (3c–2e) bond, both in its linear and triangular structures. Therefore, the energy gain when going from the linear to the triangular structure (1.76 eV) represents the ECRE, reflecting an aromatic stabilization of cyclic H_3_^+^ in S_0_ (Fig. 2). The topological analysis of the electron density also reveals a species that benefits from extensive 3c–2e bonding, manifested in the formation of a non-nuclear attractor (NNA) in the center (Fig. 4A), in agreement with previous works.^7,8^ Large DI values between the hydrogen atoms and the large positive MCI^1/*n*^ of 0.62 (Fig. 4A)^54^ are also consistent with σ-aromaticity in S_0_ (the MCI^1/*n*^ of benzene in S_0_ is 0.59 (ref. 55)). Finally, H_3_^+^ in its S_0_ state displays a diatropic MICD of 4.39 nA T^−1^, in agreement with the NICS(0)_zz_ value of −37.1 ppm, confirming the magnetic aromaticity of H_3_^+^ in S_0_ (see further Section S3 of the SI).

Evaluation of the potential antiaromaticity in the 1^1^E′ states of *D*_3h_ symmetric H_3_^+^ is more challenging from computational perspectives, requiring separate characterizations of the two states, the 2^1^A_1_ and 1^1^B_2_ states in *C*_2v_ symmetry. Indeed, our investigation offers a first exploration of whether the concept of excited-state antiaromaticity is applicable to doubly degenerate first excited states. Notably, we find that in the lowest excited state, going from the linear (*D*_∞h_) to the triangular geometry of H_3_^+^ (which is the equilibrium geometry in S_0_) results in a destabilization in the lowest vertically excited singlet states by 4.19 eV (Fig. 4B). This reveals a negative ECRE indicating antiaromaticity. Thus, the stabilization in S_0_ plus destabilization in 1^1^E′ (S_1_) upon cyclization have implications for the vertical excitation energy as their combined effects amount to 5.95 eV (Fig. 4B). The corresponding sum for the lowest triplet states is 7.50 eV. Both energies are in line with GSA-to-ESAA switches in character upon vertical excitation (Fig. 2).

Yet, the potential excited state antiaromatic character also needs to be probed through electronic and magnetic (anti)-aromaticity descriptors. With regard to the electron densities of these lowest excited states and their Laplacians, they exhibit significant differences from those of S_0_, which corroborates the loss of aromaticity upon excitation (Fig. 4A). Despite the *D*_3h_ symmetric geometry of H_3_^+^ in its vertically excited states, the electron density distribution has lower symmetry as it shifts toward the outer part of the atoms, preceding the dissociations that occur upon excitation to these states. Although the H atoms are still covalently bonded, as indicated by the value of the DIs, the 2^1^A_1_ and 1^1^B_2_ states exhibit drastic reductions of the three-center delocalization to 37–39% of the MCI value of S_0_ (Fig. 4A). Similar conclusions can be reached on the antiaromaticity of the 1^3^E′ (1^3^A_1_ and 1^3^B_2_) states based on the analysis of the electron delocalization in Fig. S2.^54^ Thus, the MCI^1/*n*^ values for H_3_^+^ (0.62 (S_0_), 0.46 and 0.45 (1^1^E′), and 0.39 and 0.36 (1^3^E′)), are comparable to those of benzene in its S_0_, S_1_, and T_1_ states (0.59, 0.40, and 0.36, respectively).^55^ This is consistent with an aromatic S_0_ state, while the lowest excited singlet and triplet states (1^1^E′ and 1^3^E′) are antiaromatic.

Going to the magnetic aspect of excited state antiaromaticity, the values of the magnetically induced ring currents obtained for the vertically excited 1^1^E′ states at the S_0_ equilibrium geometry diverge as a result of the two-fold degeneracy at *D*_3h_ symmetry. The analysis of ring current strength at the *C*_2v_ symmetric structures reveals that the 1^1^E′ states exhibit a pole in the ring current at *D*_3h_ symmetric geometries (Fig. 4C), *i.e.*, a sudden change from highly diatropic to highly paratropic values, which explains the divergence observed at the vertical excitation. This resembles recent observations on the transient antiaromatic states of the c-C_16_ molecule where small bond length alterations lead to changes from dia- to paratropicity, or *vice versa*,^57^ related to the orbital degeneracies at highly symmetric structures.^58^ Now, by following the *C*_2v_ symmetric dissociation paths of H_3_^+^, and thus, the 2^1^A_1_ and 1^1^B_2_ states, we observe gradually diminishing paratropic ring currents (Fig. 4C), in line with antiaromaticity relief. Also the NICS(0)_zz_ values computed along these paths reveal antiaromaticity, with values of 77.1 and 84.7 ppm at two *C*_2v_ symmetric structures distorted by a factor 1.5, leading to, respectively, acute and obtuse isosceles triangular structures as exemplified in Fig. 3B (see further Section S3).

### Protons-to-electrons ratios and impacts

Having established the antiaromaticity of the 1^1^E′ states of H_3_^+^, the question is if the GSA-to-ESAA switch in character (Fig. 2) explains the unusually high vertical excitation energy of this cation. From computations of light atoms, molecules and ions with only two electrons such as He, Li^+^, HHe^+^ and He_2_^2+^ (Table S3), we see that high vertical excitation energies are characteristic of these species, which mostly cannot exhibit aromaticity as they are acyclic. Many of these ions are also found in space, *e.g.*, Li^+^ and HHe^+^.^59^ Indeed, it has been argued that HHe^+^ was the first molecule of the Universe,^60,61^ and it produces H_2_^+^ upon collision with atomic H, which in turn can produce H_3_^+^ in a reaction with H_2_.^62^

The excitation energies are higher for more positively charged species, and is highest when the protons are concentrated in one nucleus (He, Li^+^ and Be^2+^). Hence, in the 3p,2e series, Li^+^ and HHe^+^ feature higher vertical *E*(S_1_) than H_3_^+^ by, respectively, 41.16 and 6.91 eV, and similar for the vertical *E*(T_1_) (Table S3). For the 2e species He, Li^+^ and Be^2+^, with protons-to-electrons ratios of 1.0, 1.5 and 2.0, respectively, the first excitation energy goes up dramatically from 20.94 eV to 60.44 eV and 121.26 eV as the excitation implies a gradually larger loss in the electrostatic attraction between electrons and nuclei.

Next, to estimate the impact of the protons-to-electrons ratio on the excitation energies, we explored the π-conjugated and S_0_ aromatic cyclopropenium cation, c-C_3_H_3_^+^, which has an equilateral triangular structure and a near-unit ratio of 1.05 between total nuclear and electronic charges (+21 *vs.* −20) (Sections S4 and S5).^63–65^ Indeed, c-C_3_H_3_^+^ is isolobal with H_3_^+^, *i.e.*, its π-orbitals are analogous to the σ-orbitals of H_3_^+^.^66^ Thus, this cation helps us decipher the relative contributions of the protons-to-electrons ratio *versus* the GSA-to-ESAA switch to the 19.28 eV excitation energy of H_3_^+^.

The vertical transition to the lowest ππ* states (1^1^E″) of c-C_3_H_3_^+^ requires 9.71 eV. This is only half that of the transition to the σσ* states of H_3_^+^ but significantly higher than the lowest ππ* states of any other π-bonded hydrocarbon, *e.g.*, 7.11 eV for ethylene.^67^ Thus, c-C_3_H_3_^+^ has its lowest ππ* states at an unusually high excitation energy despite its near-unit protons-to-electrons ratio. Interestingly, there are several σπ* and πσ* states below the first ππ* state. Therefore, the high excitation energy of the lowest ππ* transition of c-C_3_H_3_^+^ is not caused by a drastically diminished electrostatic attraction upon excitation. Instead, it should arise from the stabilization in the S_0_ state due to aromaticity plus destabilization in the ππ* states due to antiaromaticity, and this applies also to the lowest triplet states. From this, one can estimate that the additional energy (9.57 eV) to reach the excitation energy of H_3_^+^ is caused by the electrostatic effect.

Also Li_3_^+^, valence isoelectronic to H_3_^+^, is an equilateral triangle at its global minimum in the S_0_ state, but it is nonaromatic and best described as the smallest triatomic molecule with metallic bonding.^8^ We now find its lowest singlet excited states, the degenerate ^1^E states, at an energy of merely 2.70 eV. In line with the nonaromatic S_0_ state, these states are also nonaromatic (see Table S3 and Section S6 in the SI). Compression to a triangle with half the Li–Li distance leads to no substantial changes in neither the singlet excitation energy nor the electron–nucleus attraction contributions (Table S3). Among the two possible mixed Li and H monocations (H_2_Li^+^ and HLi_2_^+^), H_2_Li^+^ has an acute triangular structure with Li–H and H–H distances of 2.049 and 0.752, respectively (Fig. S7). HLi_2_^+^, on the other hand, has a linear structure^68^ and was therefore not further considered here as it is nonaromatic.

Finally, H_2_He^2+^ is also interesting in this context as it provides an electronegativity perturbation compared to the isoelectronic H_3_^+^,^59^ although our computations reveal that this dication is not a minimum on the S_0_ PES. However, when kept at the geometry which is optimal for H_3_^+^ but with one H^+^ exchanged to He^2+^, we find that it is nonaromatic in both its S_0_ state and lowest singlet excited states (Fig. S10). Accordingly, there is no GSA-to-ESAA switch in character upon the excitation of H_2_He^2+^. In line with this, there is no significant increase in excitation energy (+0.24 eV) when going from HHe^+^ to H_2_He^2+^, in contrast to what is observed when going from H_2_ to H_3_^+^ (+6.56 eV, see Table S3 and Section S6 for further discussion).

### Comparisons to analogous carbocations

**Fig. 5:**
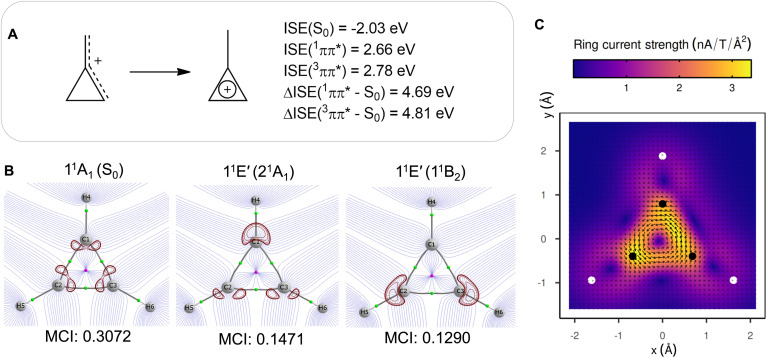
(A) Isomerization stabilization energies (ISEs) of the methylcyclopropenium cation in the S_0_ and lowest vertically excited singlet and triplet ππ* states, and the combined ISEs. (B) Topological analysis and Laplacian of the electron density (in red) of S_0_ and excited states of c-C_3_H_3_^+^ at its S_0_ geometry (1.0 a.u. above ring plane) and MCI values. (C) MICD of the cyclopropenium cation in its 1^1^E″ (1^1^B_2_) state vertically excited from S_0_ showing paratropic (antiaromatic) ring currents (1.0 a.u. above ring plane). Carbon atoms plotted as black balls and hydrogen atoms as white.

Further analysis of the c-C_3_H_3_^+^ cation, a molecular ion which also is of astrochemical importance,^29^ enables us to show that the very high first excitation energy of H_3_^+^ is due to both stabilization by GSA plus destabilization by ESAA and the high protons-to-electrons ratio. Among the (anti)aromaticity aspects, we thus first explore the energetic aspect before the electronic and magnetic ones. The isomerization stabilization energy (ISE)^69^ of c-C_3_H_3_^+^ in S_0_, computed as the reaction energy for the 1,3-hydrogen shift from a nonaromatic isomer to the S_0_ aromatic methylcyclopropenium cation (Fig. 5A), is −2.03 eV with CCSD. This reveals a stronger aromatic character than that of benzene in S_0_ (−1.34 eV with CCSD/aug-cc-pVDZ). In contrast, the lowest vertically excited singlet and triplet ππ* states exhibit large positive ISE values of 2.66 and 2.78 eV with EOM-CCSD, corresponding to strong excited state antiaromatic destabilization. Accordingly, the S_0_ stabilization plus excited state antiaromatic destabilizations of c-C_3_H_3_^+^ are 4.69 and 4.81 eV, respectively, and these should represent lower bounds for the analogous energies in H_3_^+^.

**Fig. 6:**
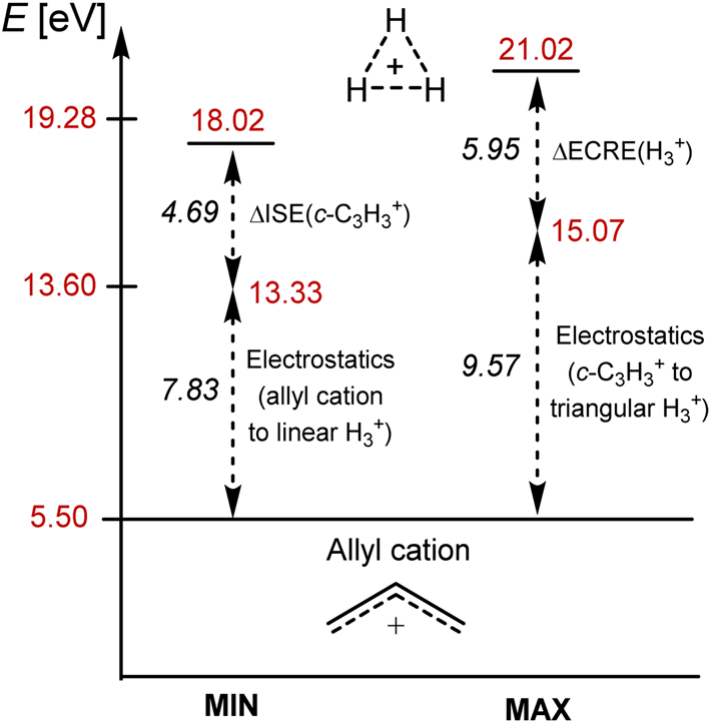
Estimation of the minimum and maximum of the excitation energy of triangular H_3_^+^ with the first ππ* excitation energy of the allyl cation as the starting point, to which energy additives representing two types of components are added: (i) the difference in electrostatics upon excitation due to different protons-to-electrons ratio in the carbocations and H_3_^+^, and (ii) the GSA-to-ESAA switch in character upon excitation. ΔISE = difference in isomerization stabilization energy between the S_0_ and lowest ππ* excited state of c-C_3_H_3_^+^, and ΔECRE = difference in extra cyclic resonance energy between the S_0_ and σσ* states of H_3_^+^.

A comparison of the various energies of the linear H_3_^+^ with the corresponding ones of the allyl cation (H_2_CCHCH_2_^+^) allows for a second estimate of the impact of the high protons-to-electrons ratio of H_3_^+^. The two species are isolobal and nonaromatic in S_0_, yet, have different protons-to-electrons counts (3 : 2 *vs.* 23 : 22). For the allyl cation, with a near-unit protons-to-electrons ratio, the lowest vertical ππ* singlet state is found at 5.50 eV, while the first σσ* excitation energy of linear H_3_^+^ is 13.33 eV, *i.e.*, 7.83 eV higher than that of the allyl cation. Both this and the excitation energy difference between c-C_3_H_3_^+^ and H_3_^+^ (9.57 eV) provide estimates of the electrostatic contribution to excitation energies of H_3_^+^ (Fig. 6).

Antiaromaticity assessments of c-C_3_H_3_^+^ in its first singlet excited ππ* state by usage of electronic and magnetic descriptors reveal a clear resemblance to H_3_^+^. The MCI values demonstrate their electronic structural similarities with the values for, respectively, the 1^1^E′ and 1^1^E″ states being less than half those of the S_0_ state (Fig. 4 and 5B), and this is further emphasized by the topological analysis of their excited state electronic structures (Fig. 4A and 5B). Finally, the MICD of the cyclopropenium cation in its lowest ππ* state reveals a paratropic (antiaromatic) ring current in the three-membered ring (Fig. 5C), similar as for the two dissociation pathways of H_3_^+^ (Fig. 4C). Furthermore, and as described above, the antiaromatic character of the lowest ππ* states, leading to an extreme destabilization, should be a strongly contributing factor to these states not being the lowest excited states of c-C_3_H_3_^+^. In contrast, for the allyl cation the lowest ππ* state is the S_1_ state. As photochemical reactions normally proceed from the lowest electronically excited state according to Kasha's rule, this will impact on the photochemistry of the c-C_3_H_3_^+^ and its alkyl substituted derivatives, likely leading to reduced photoreactivities when compared to species where ππ* states are the lowest excited states.

Thus, c-C_3_H_3_^+^ like H_3_^+^, exhibit strong aromaticity in the S_0_ state and strong antiaromaticity in, respectively, the lowest ππ* and σσ* excited states, yet the excitation energy of the latter is additionally affected by a high protons-to-electrons ratio. Indeed, by adding energy additives to the lowest excitation energy of the acyclic and nonaromatic allyl cation (5.50 eV) one can estimate the excitation energy of H_3_^+^ (Fig. 6). The energy for the GSA-to-ESAA switch in character in c-C_3_H_3_^+^ (4.69 eV) or the extra cyclic resonance energy of H_3_^+^ (5.95 eV), and the excitation energy difference between the allyl cation and linear H_3_^+^ or between triangular c-C_3_H_3_^+^ and H_3_^+^ (7.83–9.57 eV), as measures of the electrostatic energy loss between protons and electrons upon excitation, added to 5.50 eV gives 18.0–21.0 eV (Fig. 6). This energy range brackets our computed first excitation energy of H_3_^+^ of 19.28 eV. It also becomes clear that it is the GSA-to-ESAA switch in character which places the first singlet excited states well above the ionization energy of monohydrogen, and thus, provides H_3_^+^ with its astrophotochemical persistence.

## Conclusions

Herein, we decipher the main causes of the very high electronic excitation energy of H_3_^+^, which enables its functions in space. This is achieved through a comparison of triangular H_3_^+^ and linear H_3_^+^ with two analogous π-conjugated hydrocarbon ions, the cyclopropenium cation (c-C_3_H_3_^+^) and the allyl cation (CH_2_CHCH_2_^+^). Had the vertical excitation energy been lower (closer to the photoionization of the much more abundant monohydrogen), H_3_^+^ would have been more prone to photodissociate in space. We reveal that three factors contribute to the high excitation energy; (i) the change from a stabilizing aromatic character to (ii) a destabilizing antiaromatic character upon excitation from S_0_ to the lowest excited states (present also in c-C_3_H_3_^+^), and (iii) the high ratio between total nuclear and electronic charges (which is present also in small cations). These three factors together provide the origin of the astrophotochemical inertness of H_3_^+^. Without this photostability, H_3_^+^ could not have had the functions it has in the Universe, which would have led to a Universe different from the one we know. Furthermore, it may also impact on the photostability of species with similar 3-center–2-electron bonding characteristics as the H_3_^+^ ion, such as the vinyl and ethyl cations (C_2_H_3_^+^ and C_2_H_5_^+^, respectively),^29–31,70^ as well as the S_0_ state aromatic c-C_3_H_3_^+^ and derivatives, which are all of astrochemical importance.^29,70^ The excited state dynamics of these carbocationic species are not extensively explored but it can be an important direction for future research in astrochemistry.

We show for the first time that excited state antiaromaticity is a molecular electronic structure property with crucial astrochemical influence that also has astrophysical implications. In a broader sense, our findings point to the roles of excited state aromaticity and antiaromaticity as important new concepts for interpretation in astrochemistry. We anticipate that these effects impact on a number of photochemical processes in space.

## Computational methods

### Geometry and energy calculations

Geometry optimizations and energy calculations were performed with Gaussian 16 revision C.01.^71^ The electronic singlet ground state and lowest excited triplet state were calculated using CCSD while singlet excited states were computed with EOM-CCSD. For H_3_^+^, this level is equivalent to full configuration interaction (FCI), and we referred to it as FCI in the manuscript. Frequency calculations were performed at the same level of theory to probe if the structures were minima or saddle points on the potential energy surfaces. In all cases, the aug-cc-pVTZ valence triplet-zeta of Dunning and co-workers^72^ was used as the basis set. For wavefunction analysis we employed 6d 10f functions for the latter basis set. To obtain T_2_ state the orbital order was altered by usage of the guess = alter keyword in Gaussian. Calculations of electron–nucleus attraction contribution include core electrons for all molecules (see Table S3).

### Aromaticity assessment

Results of the multicentre index (MCI)^53^ were computed by usage of AIMAll,^73^ APOST-3D, and ESI-3D^56,74^ packages. Due to the presence of a non-nuclear attractor (NNA) in some of the species, we consider a different partition of the molecular space to compute the delocalization indices (DI(F))^51,52^ and the MCI. Some of us have previously found that Becke-rho's atomic partition provides similar values to partitions based on quantum theory of atoms in molecules (QTAIM),^75^ a theory which uses a topological approach to define an atom in a molecule. Becke's partition employs the position of the bond critical points (BCP) between atoms to define the atomic radii in the original Becke's partition.^76^ This multicentre integration technique assigns weights to atoms in the molecule. The calculation of the DI for correlated wavefunctions employed the so-called Fulton approximation,^77^ which provides very good agreement with the actual DI.

Isomerization stabilization energies (ISE) were computed at (EOM)-CCSD/cc-pVTZ optimized geometries but for the energy values reported we used the aug-cc-pVTZ basis set.

The magnetically induced ring current density (MICD) was calculated by employing complete active space self-consistent field (CASSCF) method (with all electrons, and occupied and virtual orbitals included in the active space, corresponding to an FCI calculation) and aug-cc-pVTZ basis set, using a development version of Dalton Program 2020.^78,79^ In order to compute the ring current passing through the bonds of the triangular H_3_^+^, the MICD was integrated in a plane perpendicular to the bond, spanning from the centre of mass of the molecule 20 bohr at opposite sides of the plane (along vectors normal to the ring plane and cutting through the bond) using 200 subdivisions in the Gauss–Lobatto quadrature.^80^

## Author contributions

Conceptualization: HO; methodology: HO, CFN, EM; investigation: JMT, JKS; visualization: JMT, JKS; funding acquisition: HO; project administration: HO; supervision: HO; analysis: JMT, JKS; writing – original draft: JMT; writing – review & editing: JMT, JKS, EM, CFN, HO.

## Conflicts of interest

There are no conflicts to declare.

## Supplementary Material

SC-OLF-D5SC09067A-s001

## Data Availability

The data underlying this study are openly available in the published article and its supplementary information (SI), and also openly available in zenodo at https://doi.org/10.5281/zenodo.15713452. The data supporting this article have been included as part of the SI. Supplementary information: Section S1: energies and geometries of H_3_^+^; Section S2: aromaticity of H_3_^+^, electronic properties; Section S3: aromaticity of H_3_^+^, magnetic properties; Section S4: protons-to-electrons ratios; Section S5: cyclopropenium cation (C_3_H_3_^+^); Section S6: Li_3_^+^, H_2_Li^+^ and H_2_He^2+^; Section S7: supplementary references; Section S8: Cartesian coordinates, absolute energies, and imaginary frequencies for all calculated structures. See DOI: https://doi.org/10.1039/d5sc09067a.
