# Supplementary material for: Deciphering the molecular origin of the 19.3 eV electronic excitation energy of H3+
Source: Chem Sci. 2026 Jan 20;17(10):5029–37. doi: 10.1039/d5sc09067a (PMC12817475; doi:10.1039/d5sc09067a)
Supplement: SC-017-D5SC09067A-s001 [file SC-017-D5SC09067A-s001.pdf]

Supplementary Information (SI) for Chemical Science.
This journal is © The Royal Society of Chemistry 2026

Electronic Supplementary Information (ESI)

Deciphering the molecular origin of the 19.3 eV electronic excitation energy of H_3^+

Josene M. Toldo,[1,2] Jakob Staab,[1,3 ‡] Eduard Matito,[4,5] Cina Foroutan-Nejad,[6] and Henrik Ottosson[1]*

[1] *Department of Chemistry - Ångström, Uppsala University, 751 20 Uppsala, Sweden;*

[2] *Université Claude Bernard Lyon 1, ENS de Lyon, CNRS, Laboratoire de Chimie, UMR 5182, 69342, Lyon cedex 07, France;*

[3] *Department of Chemistry, The University of Manchester, Oxford Road, Manchester, UK;*

[4] *Donostia International Physics Center (DIPC), 20018 Donostia, Euskadi, Spain;*

[5] *Ikerbasque, Basque Foundation for Science, 48009 Bilbao, Euskadi, Spain;*

[7] *Institute of Organic Chemistry, Polish Academy of Sciences, Warsaw, Poland.*

Table of Contents

Section S1. Energies and geometries of H_3^+ 2
Section S2. Aromaticity of H_3^+: Electronic properties 3
Section S3. Aromaticity of H_3^+: Magnetic properties 5
Section S4. Protons-to-electrons ratio 8
Section S5. Carbocations 9
Section S6. Li_3^+, H_2Li^+ and H_2He^{2+} 10
Section S7. Supplementary references 13
Section S8. Cartesian coordinates 14

‡ Present Address: *Department of Chemistry "Ugo Schiff", University of Florence, 50019 Sesto Fiorentino, Italy.*

Section S1. Energies and geometries of H_3^+

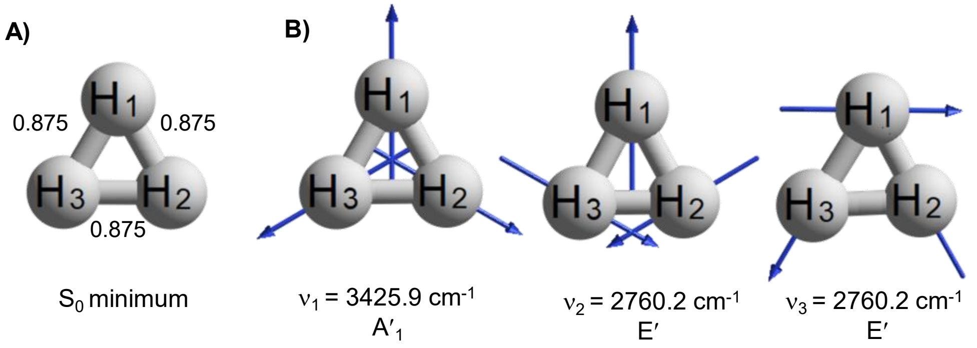

Figure S1. **(A)** Optimized geometry of H_3^+ at D_{3h} geometry and bond distances (given in Å) and **(B)** respective vibrational normal modes (in cm^{-1}). The symmetry of the vibrations are given at the D_{3h} point group. The totally symmetric ν_1 mode is infrared inactive, while the two degenerated modes ν_2 and ν_3 are active. All calculations performed at the CCSD/aug-cc-pVTZ level.

Table S1. Absolute energies (a.u.), S_1 vertical excitation energies (E_v; eV), and relative energies (ΔE; eV) calculated for H_3^+ at the $D_{\infty h}$ and D_{3h} symmetries at (EOM-)CCSD/aug-cc-pVTZ level.

	**S_0 (Hartree)**	**S_1 (Hartree)**	**E_v (eV)**	**ΔE S_0 (D_{3h} - linear) (eV)**	**ΔE S_1 (D_{3h} - linear) (eV)**
**H_3^+ $D_{\infty h}$**	-1.27710	-0.78709	13.33	1.76	4.19
**H_3^+ D_{3h}**	-1.34188	-0.63310	19.28	0.00	19.28

For the higher $1^1A_2''$ state (Figure 2A of the manuscript), earlier computational studies have located a possible shallow D_{3h} symmetric minimum at an internuclear distance of 1.61 Å on the potential energy surface (PES).[1, 2] However, according to our EOM-CCSD calculations, this point is a second-order saddle point with two imaginary frequencies (*i*562 cm^{-1}) corresponding to E′ stretch vibrations.

Section S2. Aromaticity of H_3^+: Electronic properties

Topological analysis. The Laplacian of the electron density indicates the regions of electron accumulation ($\nabla^2\rho(r) < 0$, localization) and electron depletion ($\nabla^2\rho(r) > 0$, delocalization). Although the Laplacian does not give us quantitative information about aromaticity, an aromatic molecule will show delocalisation which can be captured by looking at the isosurfaces plots.

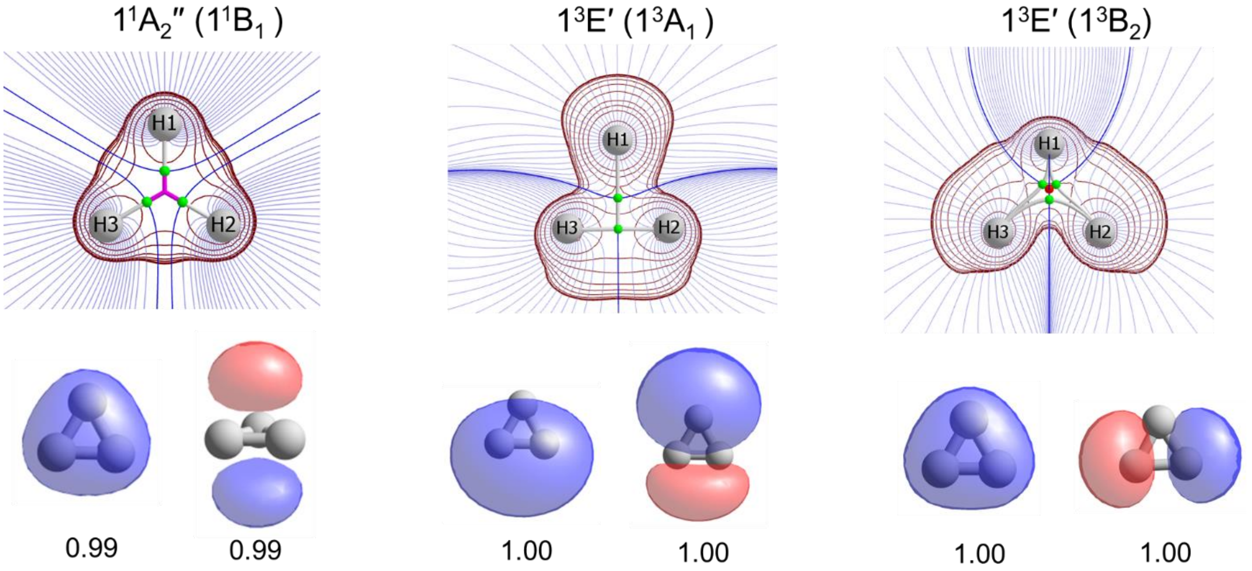

Figure S2. Topological analysis, Laplacian of the electron density (isovalue of -0.3), and natural orbitals (with respective populations) for the S_3 state (singlet $1^1A_2''$) and lowest degenerated triplets ($1^3E'$).

Delocalisation index (DI) and the multicentre index (MCI). These indices were used to identify conjugation patterns.[3, 4] The DI is a generalisation of the bond order concept and gives the number of electron pairs covalently shared between two atoms. Aromatic molecules typically exhibit DI values close to 1.5.[5] While DIs give information only about how electron density is shared covalently between two atoms, MCI values account for multicenter electronic delocalization[3, 6, 7]

and show how the electron distribution fluctuates between the atomic population of the basins. Larger MCI values suggest a higher aromatic character, whereas values at ~0 indicate nonaromatic molecules. For benzene, for example, the $MCI^{1/n}$ value in S_0 is 0.59.[8]

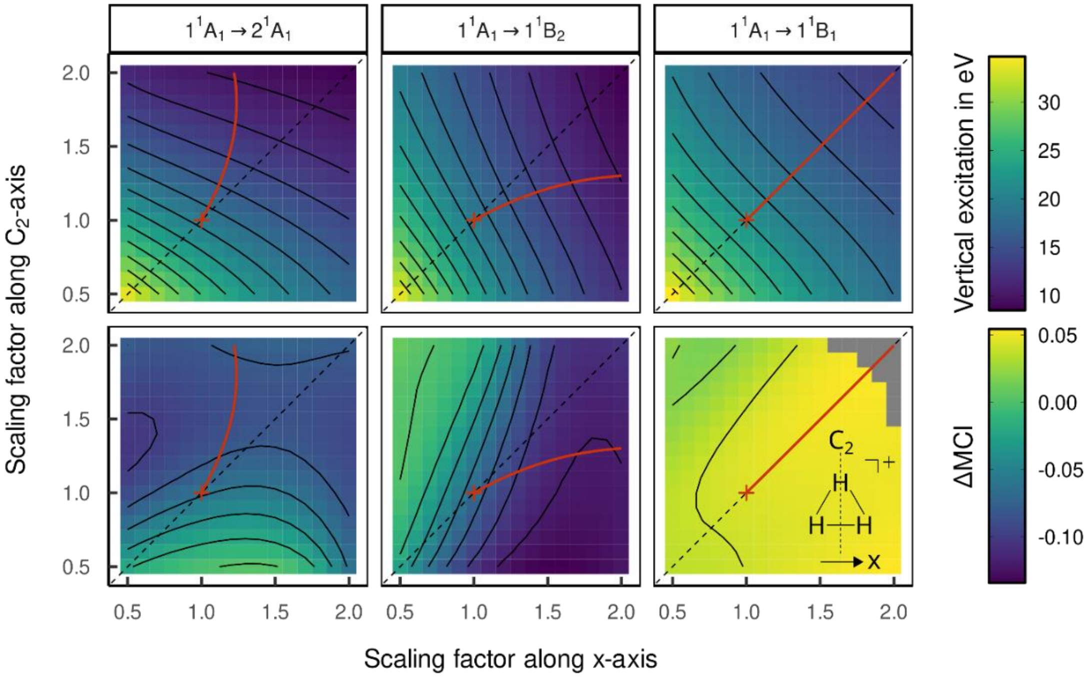

Figure S3. Vertical excitation energies and MCI values calculated when increasing the H–H interatomic distance along the totally symmetric nuclear coordinates (dashed diagonal line) and by lifting the D_{3h} degeneracy along C_2-axis and *x*-axis. The “x” in red represents the minimum energy structure of the ground state.

Table S2. Vertical excitation energies (E_v), oscillator strengths (*f*), charges (Mulliken and QTAIM), delocalization index (DI) and Multicentered index (MCI) for the ground and lowest excited singlet states of H_3^+ at D_{3h} symmetry calculated at the MS3-CASPT2//SA3-CASSCF(2,15)/cc-pVDZ level. The normalized values for MCI (calculated as $MCI^{1/n}$, where n = 3) and the original MCI values (in parenthesis). DIs and MCIs were computed using QTAIM partitioning.

State	E_v (eV)	*f*	Mulliken charges	QTAIM charges	DIs	$MCI^{1/n}$ (*MCI*)
S_0	-	-	0.33, 0.33, 0.33	0.34, 0.34, 0.34	0.38, 0.39, 0.38	0.61 *(0.228)*
2^1A_1 ($^1E' \leftarrow {}^1A_1'$)	18.77	0.56	0.29, 0.35, 0.35	0.21, 0.39, 0.40	0.27, 0.27, 0.38	0.44 *(0.086)*
1^1B_2 ($^1E' \leftarrow {}^1A_1'$)	18.77	0.56	0.37, 0.31, 0.31	0.48, 0.26, 0.26	0.29, 0.31, 0.34	0.38 *(0.053)*

Section S3. Aromaticity of H_3^+: Magnetic properties

Magnetic indices. When exposed to an external magnetic field, an aromatic molecule presents an induced electron ring current which can be used to characterize aromaticity. With the normal vector of the ring plane chosen to coincide with the *z*-axis of the coordinate system, the *zz*-component of the nuclear shielding tensor is calculated at the ring centre, commonly known as $NICS_{zz}(0)$. Negative values indicate a diatropic aromatic ring current (shielding the ring centre), and positive values indicate a paratropic ring current (deshielding the ring centre), hence antiaromaticity.

Magnetic shielding is influenced by a variety of other molecular properties, such as electron density and electron paramagnetism. However, magnetically induced ring current densities present an alternative metric which directly probes aromatic ring currents. They are calculated by integrating the current density in a plane which is delimited by the ring centre and spanned by the *z*-axis and by the vector, which passes from the origin through the middle of the

bond of two adjacent ring atoms. By convention, positive diatropic ring currents indicate aromaticity and negative paratropic ring currents are associated with antiaromaticity.

Theoretical relationship between the NICS and ring current for H_3^+ in its S_0 state. Equation 1 shows the dependence of the magnetic field B_z along the z-axis of a classical ring current I on the ring radius R at a distance z, and μ_0 is the vacuum permeability.

$$B_z = \frac{\mu_0}{2} \frac{R^2 I}{(z^2 + R^2)^{(3/2)}} \tag{1}$$

The magnetic shielding σ is defined as

$$\sigma = \frac{-B_z}{B_0} \tag{2}$$

with the external magnetic field strength given by B_0.

For diatropic ring currents, $sign(B_0) = -sign(B_z)$, combining the equations (1) and (2) leads to an expression for σ as a function of R, z and the ring current strength per applied external magnetic field (J), as shown in equation 3.

$$\sigma = \frac{\mu_0}{2} \frac{R^2}{(z^2 + R^2)^{(3/2)}} \frac{I}{B_0} = \frac{\mu_0}{2} \frac{R^2}{(z^2 + R^2)^{(3/2)}} J \tag{3}$$

This equation can be linearized, giving

$$z^2 = \left[\left(\frac{\mu_0}{2} \right)^{\frac{2}{3}} R^{\frac{4}{3}} J^{\frac{2}{3}} \sigma^{\frac{-2}{3}} \right] - R^2 \tag{4}$$

Plotting z^2 against $\sigma^{-2/3}$ (the zz-component of the shielding tensor) yields a linear relationship with a line of slope $m = \left(\frac{\mu_0}{2} \right)^{2/3} R^{4/3} J^{2/3}$ intersecting the y-axis at $b = -R^2$ (Figure S5).

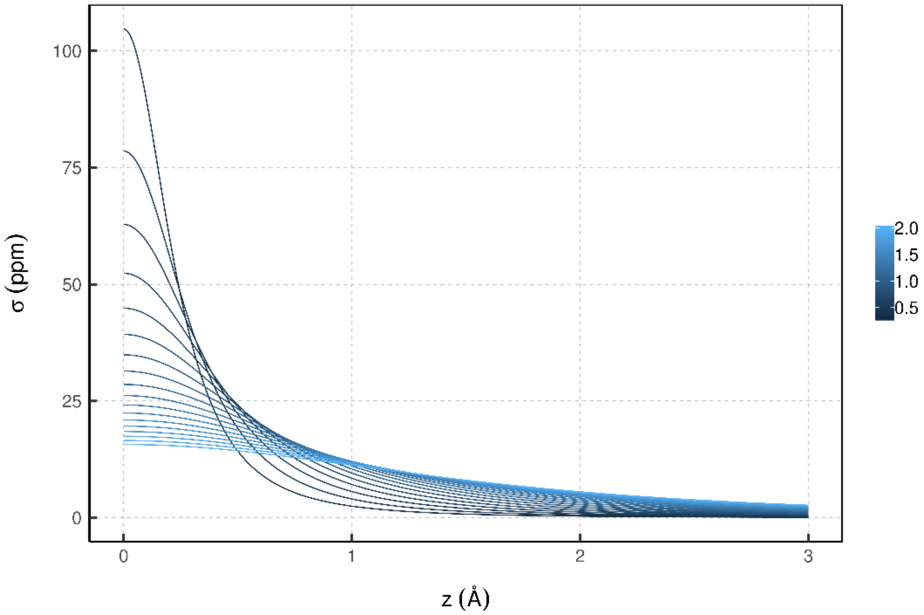

Figure S4. Theoretical ring size R (in Å) dependence of σ z-scans at a ring current of J = 5 nA T^{-1}. Small ring sizes lead to high amplitudes and curves, which decay quite rapidly at short distances.

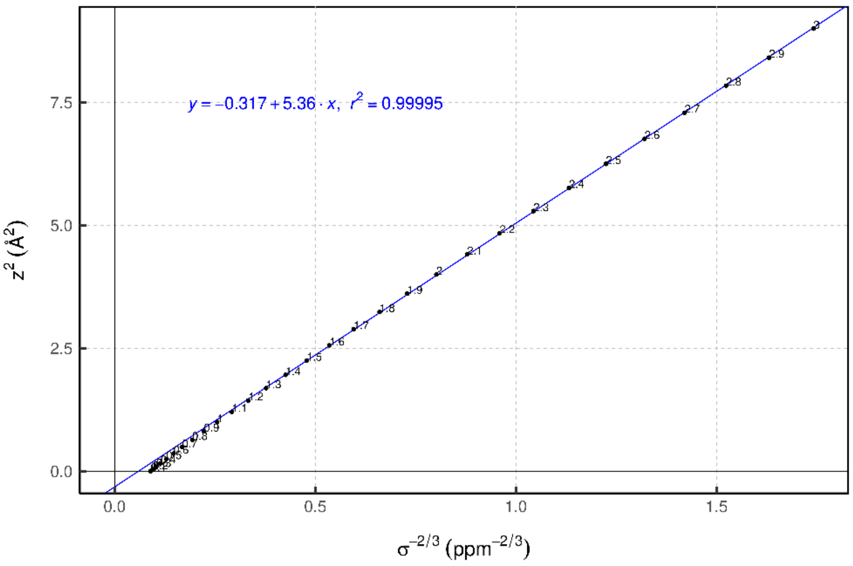

Figure S5. Correlation between z^2 and $\sigma^{-2/3}$ suggesting a direct relationship between the observed NICS values and molecular ring current. The model predicts a ring radius of R = 0.562 Å and a ring current of J = 6.23 nA.T^{-1}. The line was fitted to all points with z > 1 Å. All points are labelled with their respective z values in Å.

Section S4. Protons-to-electrons ratio

Table S3. Excitation energies and changes in nuclei-electron attraction energies. Vertical excitation energies of singlet $E(S_1)_v$ and triplet $E(T_1)_v$ states of a series of small *m*p,*n*e cations, electron-nucleus attraction contributions to these energies (ΔE_{NE}), and the $\Delta E_{NE}/E_v$ ratios. *m*p and *n*e are the number of protons and electrons, respectively. All energies in eV calculated at (EOM)-CCSD/aug-cc-pVTZ.

**Ions** (*m*p, *n*e)	$E(S_1)_v$	ΔE_{NE} $(S_0 \rightarrow S_1)$	Ratio (S_1) $\Delta E_{NE}/E_v$	$E(T_1)_v$	ΔE_{NE} $(S_0 \rightarrow T_1)$	Ratio (T_1) $\Delta E_{NE}/E_v$
1p,2e						
**H^-**	3.79	0.50	0.13	2.28	4.63	2.03
2p,2e						
**He**	20.94	56.22	2.69	19.88	56.27	2.83
**H_2**	12.72	27.05	2.13	10.57	16.59	1.57
3p,2e						
**Li^+**	60.44	154.69	2.56	58.89	152.54	2.59
**HHe^+**	26.19	36.32	1.39	21.56	31.78	1.47
**H_3^+**	19.28	21.89	1.14	14.87	13.65	0.92
4p,2e						
**Be^{2+}**	121.26	295.24	2.44	118.30	293.47	2.5
**He_2^{2+}**	34.14	20.67	0.61	19.58	-5.39	-0.28
**H_2He^{2+}** (C_{2v})[a]	26.43	22.20	0.84	19.64	19.81	1.01
9p,8e						
**Li_3^+** (D_{3h})	2.70	12.58	4.76	1.69	5.86	3.45
**Li_3^+** (D_{3h}) (×1/2)	2.74	11.22	4.17	0.65	4.58	7.14
**Li_3^+** ($D_{\infty h}$)	2.45	7.01	2.86	0.17	-4.35	-25.00
21p,20e						
**$C_3H_3^+$**	9.71	-0.03	-0.003	7.25	-2.65	-0.36

[a]This ion is unstable to dissociation; therefore, it was kept at a non-optimal geometry with the He–H and H–H distances as between the three protons in H_3^+.

Section S5. Carbocations

Table S4. Vertical excitation energies (E(S_1)$_v$, oscillator strengths (*f*), main electronic transitions and state character of *c*-$C_3H_3^+$ at its S_0 equilibrium geometry at CCSD/aug-cc-pVTZ (optimization and energies). The orbital symmetries are given according to C_{2v} point group. The fifth and sixth state are the corresponding E′ states in the D_{3h} point group.

State	E(S_1)$_v$ (eV)	*f*	Transition (coef.)	Character	Symmetry
S_1	7.60	0.000	8→12 (0.48)<br>9→11 (-0.48)	σπ*	A_2
S_2	7.78	0.000	8→12 (-0.47)<br>9→11 (-0.47)	σπ*	A_2
S_3	7.78	0.000	8→11 (-0.47)<br>9→12 (0.47)	σπ*	B_2
S_4	7.80	0.000	8→11 (-0.47)<br>9→12 (-0.47)	σπ*	B_2
S_5	9.71	0.141	10→11 (-0.64)	ππ*	A_1 (E′)
S_6	9.71	0.141	10→12 (-0.64)	ππ*	B_1 (E′)

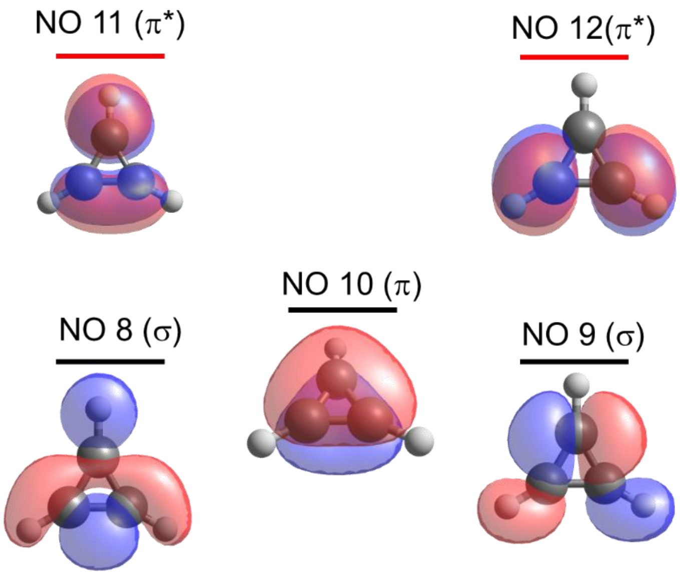

Figure S6. Natural orbitals (NO) of *c*-$C_3H_3^+$ computed with EOM-CCSD/aug-cc-pVTZ.

Table S5. Isomerization stabilization energy (ISE), *i.e.*, reaction energy of the 1,3-hydrogen shift leading to aromatic methylcyclopropenium cation from the nonaromatic isomer. Geometries computed with CCSD/cc-pVTZ and energies with (EOM)-CCSD/aug-cc-pVTZ. The wavefunction of the open-shell triplet is stable.

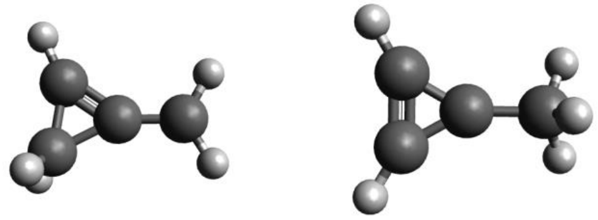

	**Nonaromatic isomer (Energy, Hartree)**	**Methylcyclopropenium cation (Energy, Hartree)**	**ISE (eV)**
**S_0 opt**	-154.7545137	-154.8291251	-2.03
**S_1 vert**	-154.6277396	-154.5613425	1.81
**S_x vert ($^1\pi\pi^*$)** [a]	-154.5965629 ($S_x = S_3$)	-154.4989167 ($S_x = S_5$)	2.66
**T_1 vertical ($^3\pi\pi^*$)**	-154.6060151	-154.5039583	2.78

[a] The first singlet ππ* state.

Section S6. Li_3^+, H_2Li^+ and H_2He^{2+}

For the Li_3^+ ion, which is known to be nonaromatic in S_0,[9] one can note much lower energies of the first singlet and triplet excited states (Table S3). The optimized geometry of Li_3^+ has long Li---Li distances of 3.004 Å (Figure S7), indicting weak bonding. As seen in Table S3, there are also no effects on the excitation energies or the ΔE_{NE} when the cluster is compressed to a structure with half the Li---Li distances (1.502 Å instead of 3.004 Å). Based on these energies there is no indication of an antiaromatic character in the lowest excited states of Li_3^+.

Moreover, the singlet excited states of both triangular and linear Li_3^+ are of low energy (2.74 and 2.46 eV, Figure S8), and the energy difference between the two structures in S_0 is much smaller than for H_3^+ (0.65 *vs*. 1.76 eV). Furthermore, in the lowest excited states the linear structure is higher than the D_{3h} symmetric one by 0.41 eV, opposite to what it should have been if the equilateral triangular structure had been antiaromatic and destabilized. These findings strengthen that also the lowest excited states of triangular Li_3^+ are nonaromatic. The analysis of the topology

of the electron density and its Laplacian is presented in Figure S9. For all states, an NNA is located at the centre of the triangular Li_3^+ structure, with bond paths becoming non-equivalent in the S_1 and S_2 excited states. Interestingly, the Laplacian of the electron density reveals patterns quite different from those found in H_3^+, with electrons localized in between the atoms – typical of a structure that lies halfway between metallic behaviour and electrides, known as *metal cluster electrides*. This reinforces the non-aromatic character of all the states of Li_3^+.[10]

The H_2Li^+ ion adopts a strongly acute triangular geometry which is best described as a Li^+ cation weakly coordinated to an H_2 molecule (Figure S7, right panel). The H-H bond length in H_2Li^+ is essentially unchanged compared to that of H_2, and the Li---H distances are very long. It is apparent that this ion exhibits no σ-aromaticity in its S_0 state, even though it has two valence electrons. For this reason, we did not carry out any additional aromaticity analysis and we also did not explore its lowest electronically excited states.

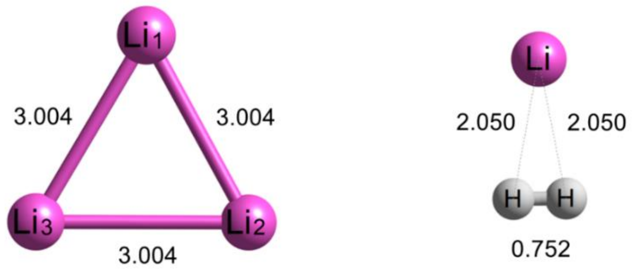

Figure S7. Optimized D_{3h} symmetric geometry of Li_3^+ in S_0 and the C_{2v} symmetric geometry of LiH_2^+ in S_0 calculated at CCSD/aug-cc-pVTZ level. Bond distances given in Å.

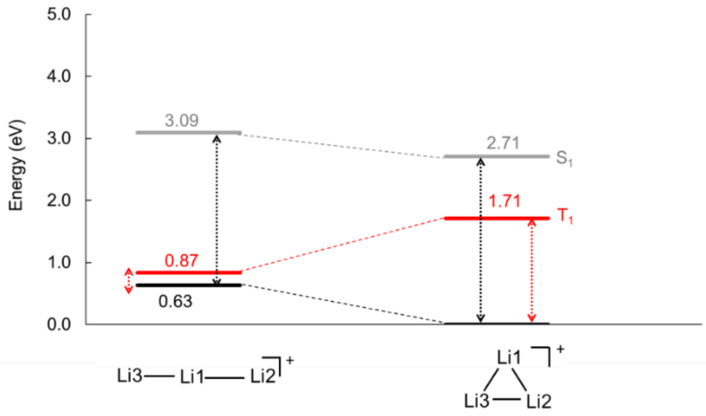

Figure S8. Energies of S_0, T_1 and S_1 computed for Li_3^+ at linear and D_{3h} structure in the ground state computed at (EOM-)CCSD/aug-cc-pVTZ level (optimization and energies).

Li_3^+

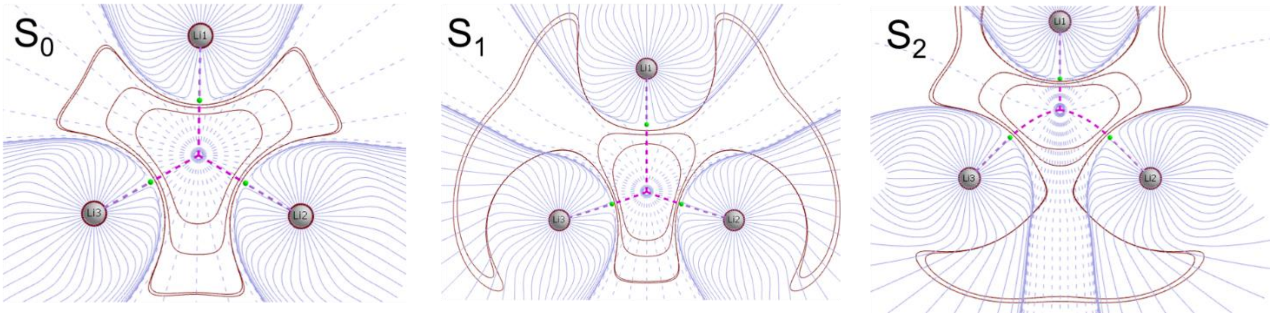

Figure S9. Topological analysis and Laplacian of the electron density (in red) of S_0 and the lowest singlet excited states of Li_3^+ at its optimal S_0 state geometry.

Finally, we investigated the hypothetical H_2He^{2+} dication, which is isoelectronic with H_3^+, but with a strong electronegativity perturbation due to the He atom. When going from diatomic HHe^+ to the triatomic H_2He^{2+} one can note that the increase in the lowest singlet excitation energy is very modest (+0.24 eV, Table S3). Of these two species, the first is a nonaromatic diatomic ion while the second potentially can be aromatic in S_0 and antiaromatic in S_1 and T_1. However, the negligible excitation energy increase when going from HHe^+ to H_2He^{2+} indicates that there is no increase on its first excitation energy due to a GSA-to-ESAA switch in character in the latter species. When going from the diatomic H_2 to the triatomic H_3^+, on the other hand, there is a large increase in energy (6.56 eV, Table S3). Similar observations can be made in the T_1 state because a decrease in excitation energy of 1.92 eV is observed when going from HHe^+ to H_2He^{2+}, whereas an increase of 4.30 eV is found when moving from H_2 to H_3^+. This contrasting behavior reinforces that a GSA-to-ESAA change in character occurs in the equilateral triangular hydrogen species (Table S3) but not in the helium-containing ones. Additionally, the MCI value of H_2He^{2+} in S_0 (Figure S10) is far below the value that computed for H_3^+ (0.228, Table S2). The analysis of the topology of the electron density and its Laplacian presented in Figure S10 shows that the topology changes substantially from one state to another. In particular, the Laplacian indicates that electrons are delocalized along the triangular structure of S_0, consistent with the large MCI value, whereas neither S_1 and S_2 exhibit significant delocalization between He and the hydrogen atoms, in line with the marked reduction of MCI in these excited states.

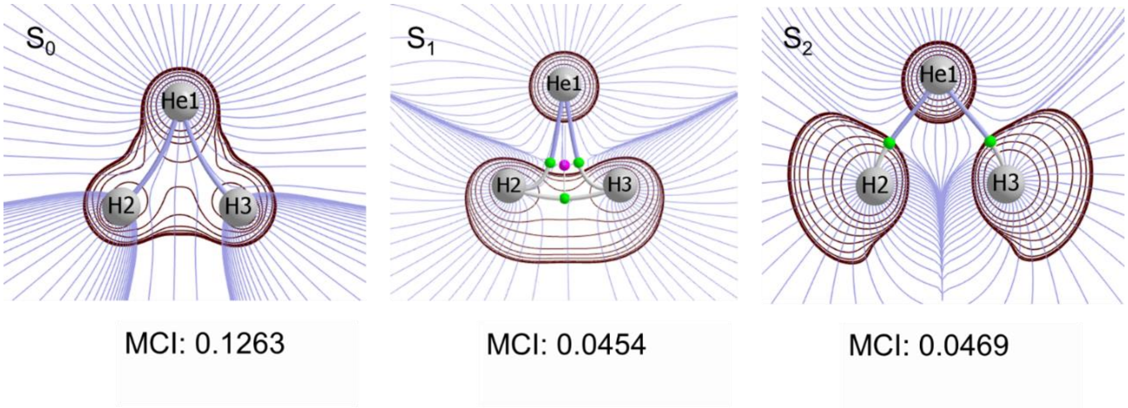

Figure S10. Topological analysis and Laplacian of the electron density (in red) of ground and excited states of H_2He^{2+}. This ion is unstable, therefore, it was kept at a frozen geometry with both the He–H and H–H distances equal to the H–H distances in the optimal geometry of H_3^+ in S_0. MCI for each state value are shown as inserts in the figure. All calculations were done at (EOM-)CCSD/aug-cc-pVTZ.

Section S7. Supplementary references

1. K. Kawaoka and R. F. Borkman, *J. Chem. Phys.*, 1971, **54**, 4234-4238.
2. H. Conroy, *J. Chem. Phys.*, 1969, **51**, 3979-3993.
3. P. Bultinck, R. Ponec and S. Van Damme, *J. Phys. Org. Chem.*, 2005, **18**, 706-718.
4. J. Cioslowski, E. Matito and M. Solà, *J. Phys. Chem. A*, 2007, **111**, 6521-6525.
5. X. Fradera, M. A. Austen and R. F. W. Bader, *J. Phys. Chem. A*, 1999, **103**, 304-314.
6. P. Bultinck, M. Rafat, R. Ponec, B. Van Gheluwe, R. Carbó-Dorca and P. Popelier, *J. Phys. Chem. A*, 2006, **110**, 7642-7648.
7. A. M. K. Müller, *Phys. Lett. A*, 1984, **105**, 446-452.
8. F. Feixas, J. Vandenbussche, P. Bultinck, E. Matito and M. Solà, *Phys. Chem. Chem. Phys.*, 2011, **13**, 20690-20703.
9. C. Foroutan-Nejad and P. Rashidi-Ranjbar, *J. Mol. Struct. THEOCHEM*, 2009, **901**, 243-248.
10. O. El Bakouri , V. Postils, M. Garcia-Borràs, M. Duran, J.M. Luis, S. Calvello, A. Soncini, E. Matito, F. Feixas, and M. Solà, *Chem. Eur. J.*, 2018, **24**, 9853-9859

Section S8. Cartesian coordinates

Table S6. Cartesian coordinates, absolute energies, and imaginary frequencies for H_3^+ structures.

```
3
H3(+) D3h S0 state (CCSD/aug-cc-pVTZ)
H        0.000000000      0.505121000      0.000000000
H        0.437448000     -0.252561000      0.000000000
H       -0.437448000     -0.252561000      0.000000000
E(S0): -1.3418815035; 0 imaginary frequencies
```

```
3
H3(+) linear S0 state (CCSD/aug-cc-pVTZ)
H        0.000000000      0.000000000      0.815944000
H        0.000000000      0.000000000      0.000000000
H        0.000000000      0.000000000     -0.815944000
E(S0): -1.2770961862; 2 imaginary frequencies (-1527.12; -1527.12)
```

```
3
H3(+) linear T1 state (CCSD/aug-cc-pVTZ)
H        0.000000000      0.000000000      1.298768000
H        0.000000000      0.000000000      0.000000000
H        0.000000000      0.000000000     -1.298768000
E(T1): -1.1155419821; 0 imaginary frequencies
```

Table S7. Cartesian coordinates, absolute energies, and imaginary frequencies for $C_3H_3^+$ and allyl cation, both in the S_0.

```
6
C3H3(+) D3h S0 state (CCSD/aug-cc-pVTZ)
C        0.000000000      0.786298000      0.000000000
C       -0.680954000     -0.393149000      0.000000000
C        0.680954000     -0.393149000      0.000000000
H        0.000000000      1.865152000      0.000000000
H       -1.615269000     -0.932576000      0.000000000
H        1.615269000     -0.932576000      0.000000000
E(S0): -115.27824105; 0 imaginary frequencies
```

```
8
Allyl cation S0 state (CCSD/aug-cc-pVTZ)
C        0.000000000      0.519454000      0.000000000
H        0.000000000      1.599988000      0.000000000
C       -1.177825000     -0.197377000      0.000000000
H       -1.164888000     -1.281986000      0.000000000
H       -2.144949000      0.292684000      0.000000000
C        1.177825000     -0.197377000      0.000000000
H        2.144949000      0.292684000      0.000000000
H        1.164888000     -1.281986000      0.000000000
E(S0): -116.72631489; 0 imaginary frequencies
```

Table S8. Cartesian coordinates, absolute energies, and imaginary frequencies for aromatic methylcyclopropenium cation and its nonaromatic isomer.

```
9
methylcyclopropenium cation S0 state (CCSD/cc-pVTZ)
C       -1.071391000     -0.677678000      0.000969000
C       -1.071681000      0.677542000      0.000979000
C        0.120479000      0.000212000     -0.010121000
H       -1.621102000     -1.604748000      0.002669000
H       -1.621781000      1.604382000      0.002699000
C        1.587889000      0.000221000     -0.006341000
H        1.904039000     -0.008729000      1.041059000
H        1.973129000     -0.895179000     -0.486641000
H        1.973939000      0.902491000     -0.472691000
E(S0, CCSD/cc-pVTZ): -154.74630128; 0 imaginary frequencies
```

```
9
Nonaromatic methylcyclopropenium cation  isomer S0 state (CCSD/cc-pVTZ)
C        0.795278000      0.818441000      0.000040000
H        1.177797000      1.830071000     -0.000400000
C       -0.263641000     -0.026690000      0.000030000
C       -1.617781000     -0.086701000     -0.000110000
H       -2.198232000      0.828139000      0.000660000
H       -2.141111000     -1.034841000     -0.000330000
C        1.108359000     -0.625709000      0.000020000
H        1.514359000     -1.049658000     -0.915650000
H        1.513899000     -1.049758000      0.915850000
E(S0, CCSD/cc-pVTZ): -154.67210827; 0 imaginary frequencies
```

Table S9. Cartesian coordinates, absolute energies, and imaginary frequencies for Li_3^+ D_{3h}, Li_3^+ linear, HeH_2^{2+}, and LiH_2^+.

```
3
Li3(+) D3h S0 state (CCSD/aug-cc-pVTZ)
Li       0.000000000      1.734551000      0.000000000
Li       1.502165000     -0.867275000      0.000000000
Li      -1.502165000     -0.867275000      0.000000000
E(S0, CCSD/aug-cc-pVTZ): -22.207500792; 0 imaginary frequencies
```

```
3
Li3+ linear S0 state (CCSD/aug-cc-pVTZ)
Li       0.000000000      0.000000000      3.058570000
Li       0.000000000      0.000000000      0.000000000
Li       0.000000000      0.000000000     -3.058570000
E(S0, CCSD/aug-cc-pVTZ): -22.184620773; 2 imaginary frequencies
(-53.3115; -53.3115)
```

```
3
HeH2(2+)S0 state (CCSD/aug-cc-pVTZ)
He       0.000000000      0.000000000      0.378841000
H        0.000000000      0.437448000     -0.378841000
H        0.000000000     -0.437448000     -0.378841000
E(S0, CCSD/aug-cc-pVTZ): -2.4806780206
```

Single point energy at a geometry frozen to that corresponding to the optimal D3h symmetric geometry of H_3^+ but with one H^+ exchanged to He^{2+}.

```
3
LiH2(+) C2v S0 state (CCSD/aug-cc-pVTZ)
Li       0.000000000      0.000000000      0.806052000
H        0.000000000      0.375837000     -1.209078000
H        0.000000000     -0.375837000     -1.209078000
E(S0, CCSD/aug-cc-pVTZ): -8.4183296914; 0 imaginary frequencies
```
